# Patients' perspectives on buprenorphine subcutaneous implant: a case series

**DOI:** 10.1186/s13256-024-04483-6

**Published:** 2024-04-06

**Authors:** Claudio Pierlorenzi, Marco Nunzi, Sabino Cirulli, Giovanni Francesco Maria Direnzo, Lucia Curatella, Sandra Liberatori, Annalisa Pascucci, Edoardo Petrone, Generoso Ventre, Concettina Varango, Maria Luisa Pulito, Antonella Varango, Cosimo Dandolo, Brunella Occupati, Roberta Marenzi, Claudio Leonardi

**Affiliations:** 1 UOS Patologie da Dipendenza D9 ASL Roma 2, Rome, Italy; 2 UOC Patologie da Dipendenza D8 ASL Roma 2, Rome, Italy; 3 UOS Terapia del Dolore ASL Roma 2 Ospedale S. Eugenio, Rome, Italy; 4 Servizio Dipendenze Casalpusterlengo, ASST Lodi, Lodi, Italy; 5 Tossicologia Medica, Azienda Ospedaliero Universitaria Careggi, Florence, Italy; 6 ASST Papa Giovanni XXII, Ospedale Di Bergamo, Bergamo, Italy; 7 Dipartimento Tutela Delle Fragilità ASL Roma 2, Rome, Italy

**Keywords:** Buprenorphine implant, Opioid agonist therapy, Opioid use disorder, Sublingual buprenorphine

## Abstract

**Background:**

Considering the enormous burden represented by the opioid use disorder (OUD), it is important to always consider, when implementing opioid agonist therapy (OAT), the potential impact on patient’s adherence, quality of life, and detoxification. Thus, the purpose of the study is to evaluate how the introduction of a novel OAT approach influences these key factors in the management of OUD.

**Case presentation:**

This article marks the pioneering use of OAT through buprenorphine implant in Europe and delves into the experience of six patients diagnosed with OUD at a relatively young age. The patients, comprising both males and a female, are of Caucasian Italian and African Italian ancestry (case 4) and exhibit an age range from 23 to 63, with an average drug abuse history of 19 ± 12 years. All patients were on stable traditional OAT before transitioning to buprenorphine implants. Despite the heterogeneity in social and educational backgrounds, health status, and drug abuse initiation histories, the case series reveals consistent positive treatment outcomes such as detoxification, absence of withdrawal symptoms and of side effects. Notably, all patients reported experiencing a newfound sense of freedom and improved quality of life.

**Conclusions:**

These results emphasise the promising impact of OAT via buprenorphine implants in enhancing the well-being and quality of life in the context of OUD.

**Supplementary Information:**

The online version contains supplementary material available at 10.1186/s13256-024-04483-6.

## Introduction

Opioid use disorder (OUD) is a chronic, relapsing condition accounting for over 16 million people worldwide [1, 2]. International guidelines recommend opioid agonist therapy (OAT) with sublingual buprenorphine or methadone as first-line treatments of opioid dependence [3]. However, the rates of oral OAT misuse, abuse, and diversion are of public concern due to their social, sanitary, and economic repercussions [4, 5]. Additionally, patient adherence to oral OAT remains a challenge nowadays.

Little research has been carried out about strategies to support long-term remission from opioid dependence [6, 7]. An implantable formulation of buprenorphine has been developed to address problems with adherence, diversion, and non-medical use [6]. The rod-shaped implant consists of a mixture of a polymeric ethylene vinyl acetate matrix and buprenorphine that, following an initial pulse release, delivers a constant and stable medication level over 6 months after a single procedure [8].

Buprenorphine implant has shown its effectiveness in placebo-controlled studies [6, 8, 9] displaying a significant reduction of the opioid abuse (percentage of opioid-negative urine samples: 36% in implant group vs. 14.4% in placebo) and percentage of participants who completed the study [8]. As compared with standard sublingual buprenorphine or buprenorphine/naloxone tablets, the implant showed comparable efficacy and adverse event rate [6, 8]. A systematic benefit-risk assessment, based on a semiquantitative analysis of the available data, found a favourable profile for buprenorphine implant in comparison to sublingual buprenorphine [10]. The main benefits identified for buprenorphine implant included: improved compliance and convenience, reduced risk of illicit opioid abuse, quality of life, and risk of misuse/diversion. On the other hand, risks were mostly associated with the insertion and removal procedure. The benefits mentioned so far outweighed the risks [10]and long-acting buprenorphine implants appears to sustain the long-term remission of patients suffering from OUD [10].

This article describes a series of patients with OUD who received OAT through buprenorphine implant, marking the pioneering cases at the European level. Each case report provides a comprehensive narrative, encompassing the patients' history and clinical progression, starting from the initiation of drug abuse to the subsequent outcomes (in terms of detoxification, absence of withdrawal symptoms, side effects and improved quality of life) achieved with buprenorphine implant.

## Case report 1

### Clinical case description

The patient is a 54-year-old male of Caucasian Italian ancestry with lower secondary education. The patient, the youngest child of 5, experienced the tragic loss of a brother at the age of 17 due to an accident, and the father passed away 38 years ago from gastric haemorrhage. The mother, who is still alive and in fair health, works from home as a seamstress. The patient lives with his mother, but often sleeps away from home because of work. He engaged in a romantic relationship, including cohabitation, which lasted for a few years. Ultimately, at the conclusion of this period, he returned to live with his mother and stated: “*I was not in the right state of mind… Who wants to be with me? I'm never at home… and then I'm fine like this*”. He worked as a welder for a brief period. At the age of 18, he started working as a courier and then as a truck driver for third parties, constantly moving around Italy. Currently, he continues to work as a truck driver, but on his own account.

#### Medical history

The patient reports having contracted common childhood exanthems and undergoing a splenectomy due to a car accident in his 20s, followed by hemotransfusions. In 1986, he was diagnosed with chronic HCV hepatitis (it is unclear whether it was related to drug addiction), classified as G4, F2-related, and was treated with glecaprevir/pibrentasvir.

#### Toxicological history

At the age of 23, the patient began his journey with drugs by abusing intravenous heroin, cocaine, and alcohol (in the latter case, moderately). He was referred in April 1991, based on Article 75, to the Addiction Service of Lodi by the Prefecture of Piacenza. Two months later, the patient started OAT with a daily dose of 50 mg of methadone. From the age of 23 to 27, the patient exhibited very oppositional behaviour: he was lying, provocative, sometimes aggressive and threatening. During that period, the patient began and interrupted several therapeutic programmes.

### Traditional opioid agonist therapy

In July 2004, the first contact with our Addiction Service occurred. The patient began therapy with sublingual buprenorphine at 8 mg/day in increments, but he never completed the scaling. In this regard, in 2013, we read in the clinical diary: *“He is not able to disengage from buprenorphine despite remaining abstinent from drugs for some months”*. The patient continued with sublingual buprenorphine 2 mg/day until May 2018, at which point he transitioned to a dosage of 2 mg every other day. The patient maintained this regimen until June 2022.

### Buprenorphine implant

In May 2022 we proposed the subcutaneous buprenorphine implant treatment to the patient, as he appeared to align with the characteristics of the ideal patient. He showed immediate interest and accepted. The selection was based on his consistent use of 2 mg sublingual buprenorphine every other day over the years, prolonged negative drug tests, frequent business-related travel as a lorry driver, and the logistical challenge of attending the Addiction Service every weekend (which also involved transfers to various Services). Furthermore, the patient expressed a desire to avoid encounters with other users at the Addiction Service with whom he no longer wished to share experiences.

In August 2022, the implant surgery was conducted for the patient.

#### Follow up visits

Throughout the six months of treatment, the patient underwent several visits, including monthly and sometimes fortnightly follow-ups. A urine toxicology check was performed every two weeks, consistently yielding negative results. The patient did not encounter any issues with the implanted arm site, finding it easy to use. He reported a notable absence of the fluctuations ("spikes") experienced with tablet intake, a diminished taste for cigarettes, and a complete lack of cravings for drugs. He expressed satisfaction with his choice but recommended the buprenorphine implant primarily for individuals aiming to cease the use of drugs of abuse. In his perspective, the implant may seem "a bit light" and more suitable for those seeking complete abstinence rather than those intending to remain on agonist therapy. The patient did not have interviews with the psychologist due to work-related commitments.

## Conclusions

The organisation and management of the patient’s surgery proceeded smoothly. The patient was consistently monitored through visits, urine tests, and phone calls, especially during his business travels. The psychophysical condition of the patient has always been good, and the patient also observed a stabilisation in his nightly rest. In February 2023, the patient removed the device after the 6-month period, expressing great satisfaction with the experience. Subsequently, the patient did not encounter any issues and did not require buprenorphine/naloxone. In fact, the patient conveyed the intention to abstain from a second implant and forgo further OAT because he felt well. During the months with the implant, he successfully distanced himself from addiction after many years.

## Case report 2

### Clinical case description

The patient is a 63-year-old man of Caucasian Italian ancestry who underwent treatment at the Medical Toxicology Department in Florence. He is a former addict, having maintained abstinence for over 30 years from heroin and methadone. After an extended period of traditional OAT with sublingual buprenorphine, he consistently expressed his desire to discontinue this treatment. Subsequently, the patient was presented with the option of a buprenorphine implant, which he accepted with the goal of achieving detoxification as the dosage in the subcutaneous implant is depleted by the end of the 6th month.

The patient's family history includes a hypertensive mother who died in 2010at the age of 86, a father who died at the age of 89, and an older sister in apparent good health. Throughout his life, the patient has experienced chronic hypoxia, maintained a low body mass index (BMI), and displayed regular diuresis and bowel function. Employed as an office worker, he grapples with insomnia and smokes approximately 15 cigarettes daily. Since the 1990s, the patient has tested positive for Hepatitis C (HCV). In 2008, he was diagnosed with renal heteroplasia on the right side, necessitating surgical exeresis. In 2010, a fracture of the right distal condyle of the femur occurred, prompting surgical intervention. From 2017 onward, the patient has been under the surveillance of the Systemic Manifestations of Hepatitis Virus Centre (MASVE), where he was diagnosed with cryoglobulinemia. Successful HCV eradication measures were undertaken.

#### Toxicological history

The patient began illicit drug abuse in 1978 at the age of 19, with heroin being the primary substance of abuse. Of note, around the age of 30, the patient underwent a period of community day care. Concomitantly, he has consistently used and continues to use cannabinoids. Currently, the patient has been abstinent from heroin use for about 30 years.

### Traditional opioid agonist therapy

From 1982 to 2007, the patient received treatment at the Medical Toxicology Department of the regional reference centre with methadone for heroin use disorder. Subsequently, he underwent OAT with sublingual buprenorphine until October 2022 (Table 1), at which point he transitioned to buprenorphine implant therapy.

**Table 1:** Sublingual buprenorphine therapy

Since:	to:	Dosage
2007	2010	4 mg
2010	2016	6 mg
2016	2017	8 mg
2017	2019	10 mg
2019	2021	12 mg
2021	2022	6 mg
September 2022	October 2022	8 mg

#### Psychological aspects prior to buprenorphine implant

The patient exhibits compensated histrionic traits without psychosocial relapse. He is also characterised by an anxious temperament but maintains an on-axis mood [8]. The acceptance of this treatment stems from the desire for increased freedom, as it eliminates the need for frequent visits to the facility for sublingual buprenorphine, with the ultimate goal of achieving definitive detoxification.

### Buprenorphine implant

At the time of implantation, the patient was on 8 mg sublingual buprenorphine agonist therapy. The patient underwent subcutaneous implant surgery in October 2022. The implantation was performed at the Vascular Access Centre Unit, Department of Anaesthesia and Resuscitation AOUC (for a comprehensive outline of the procedure, please refer to Additional file 1: Appendix SI). Except for the initial days when the patient experienced mild withdrawal symptoms and a minor infection at the implant site, promptly addressed with antibiotics, the patient expressed overall satisfaction and happiness with the decision made.

#### Follow-up visits

The patient engaged in numerous follow-up visits, during which evaluations were performed to assess both physical and psychological outcomes. The Clinical Opiate Withdrawal Scale (COWS) score was employed throughout these visits to monitor the patient's withdrawal symptoms and general well-being (Table 2). The COWS categorical score ranges are defined as follows: no withdrawal (0–4), mild (5–12), moderate (13–24), moderately severe (25–36), and severe withdrawal (> 36) [11, 12].

**Table 2:** Main outcomes of the follow-up visits

Follow-up visit	Physical and psychological examination	COWS score and intervention
*24 h after the implant*	• The patient reported a subjective feeling of overdose and headache • The physical examination revealed hypertension with a BP reading of 160/100 mmHg	• COWS score = 0 • The patient reported no craving
*Day 5 after the implant*	• The wound at the implant site was found to be in good condition, with slight bruising on the repositioned fourth rod site • Amoxicillin and clavulanic acid 875 mg/125 mg were prescribed at a dosage of one tablet twice a day	• COWS score = 5 • The patient experienced mild withdrawal symptoms with no significant cravings • Treatment with 1 mg SL buprenorphine for 3 days was effective in alleviating the withdrawal symptoms
*Day 11 after the implant*	• The patient showed good toxicological compensation and was overall satisfied with the choice • The patient was still hypertensive with a BP of 145/90 mmHg	• COWS score = 0 • The patient reported no craving
*Day 16 after the implant*	• A good toxicological compensation was confirmed, and the patient expressed overall satisfaction with the subcutaneous implant • The urine test was negative	• COWS score = 0 • The patient reported no craving
*1.5 to 7 months after the implant*	• The patient presented with on-axis mood, no free anxiety, a hypnic pattern within limits, and restorative sleep • The urine test was negative	• COWS score = 0 • The patient reported no craving

*BP* blood pressure

*COWS* clinical opiate withdrawal scale

*SL* sublingual

The removal of the implant, initially planned at the latest after 7 months from insertion, was delayed by a few months at the patient's request. The patient underwent monitoring of buprenorphine blood levels, which showed a slow decline in values, maintaining excellent toxicological compensation. The removal procedure was scheduled for the July 17, 2023, at the Vascular Access Centre Unit of the AOUC, but it was unsuccessful. After 2 h, the removal intervention was interrupted, and the patient was directed to ultrasound and MRI examination, which allowed visualization of the implants in the subfascial space in the brachial biceps muscle of the left arm instead of subcutaneous space. Following a thorough orthopaedic consultation, it was decided to forgo surgical intervention due to the patient's asymptomatic clinical presentation. Instead, the plan is to monitor the progress through semi-annual follow-ups. As of now, no complications have been identified.

## Conclusions

The patient consistently reported minimal withdrawal symptoms and no significant cravings throughout the follow-up period with an excellent toxicological compensation. Furthermore, the patient expressed overall satisfaction with the subcutaneous implant, emphasizing its positive impact on mood, anxiety levels, and sleep patterns. Despite the initial challenges in the removal procedure, the patient's clinical presentation remains asymptomatic, contributing to the overall success of the buprenorphine implant treatment.

## Case report 3

### Clinical case description

The patient, a 55-year-old woman of Caucasian Italian ancestry, was admitted to a psychiatric clinic in 2012 with a diagnosis of “depressive syndrome in a patient suffering from bipolar disorder, diffuse polyarthralgias and resumption of alcoholism”. She has been consistently under the care of a trusted psychiatrist since then.

#### Toxicological history

Her primary substance of abuse was heroin until the late 1990s, followed by the development of alcohol use disorder. Alcohol consumption persisted over the years with long periods of remission and brief relapses mainly in a binge-like manner. Due to her history, the patient had been actively engaging with the Alcohol Centre and participating in self-help groups. She has been abstinent from alcohol consumption since 2021 and from heroin for over 20 years.

### Traditional opioid agonist therapy

Since 2004, the patient has been undergoing OAT with buprenorphine (Table 3), and during this period, she has also been consistently receiving stable and concurrent psychopharmacological therapy. The patient had repeatedly expressed interest in discontinuing OAT, thus at the end of 2022 she was offered the option of using a buprenorphine implant. The proposed plan involved utilizing the implant for a duration of either 6 or 12 months, contingent on the patient's decision to pursue or decline a second implant at the conclusion of the initial period. This approach aimed to facilitate the detoxification process. At the time of the decision, the patient was in good compensation from a psychiatric and toxicological point of view.

**Table 3:** Sublingual buprenorphine therapy

Since:	To:	Dosage
2004	2008	bup—8 mg
2008	Dose reduction attempted	bup—1mg
2009	2012	bup—8 mg
2012	2014	bup + nal—8 mg
2014	Dose reduction attempted	bup + nal—6 mg
2014	2016	bup + nal—12 mg
2016	2017	bup + nal—8 mg
2017	Dose reduction attempted	bup + nal—4 mg
2018	2019	bup + nal—8 mg
2019	2020	bup + nal—12 mg
2020	2021	bup + nal—8 mg
2021	2023	bup + nal—12 mg
January 2023	Dose reduction	bup + nal—8 mg

*Bup* buprenorphine

*bup + nal* buprenorphine + naloxone

### Buprenorphine implant

The patient underwent subcutaneous implant surgery in February 2023. For the detailed procedure, please refer to Additional file 1: Appendix SII. The patient did not show any signs of withdrawal or overdose in the days following implantation.

#### Psychological aspects following the buprenorphine implant

Generally, the patient considers herself satisfied and happy with the choice made. Moreover, the World Health Organization Quality of Life – BREF (WHOQOL-BREF), a self-report questionnaire assessing quality of life [13], was administered to the patient. Her assessment yielded the following scores: physical health = 21 (scale range: 7–35), psychological health = 23 (scale range: 6–30), social relationships = 10 (scale range: 3–15), and environment = 27 (scale range: 8–40).

#### Follow-up visits

During the follow-up visits, the patient’s physical and psychological state were assessed, and the COWS score was employed to evaluate withdrawal symptoms and general well-being (Table 4).

**Table 4:** Main outcomes at the follow-up visits

Follow-up visit	Physical and psychological examination	COWS score and intervention
*24 h after the implant*	• No distress reported • BP of 120/75 mmHg	• COWS = 0 • The patient reported no craving
*Day 7 after the implant*	No distress reported	• COWS = 0 • The patient reported no craving
*Day 15 after the implant*	Daytime drowsiness and diarrhoea reported	• COWS = 0 • The patient reported no craving
*1.5 months after the implant*	No distress reported	• COWS = 0 • The patient reported no craving
*3 months after the implant*	No distress reported	• COWS = 0 • The patient reported no craving
*4.5 months after the implant*	No distress reported	• COWS = 0 • The patient reported no craving
*6 months after the implant*	No distress reported	• COWS = 1, grading 1 • The patient reported no craving but was administered 2mg SL buprenorphine to alleviate her anxiety
*7 months after the implant*	No distress reported	• COWS = 0 • The patient reported no craving but was administered 4mg SL buprenorphine to alleviate her anxiety

*BP* blood pressure

*COWS* clinical opiate withdrawal scale

*SL* sublingual

## Conclusions

Throughout the observation period, the patient displayed overall well-being. However, as the removal procedure approached, she experienced mild anxiety, which was successfully managed with low doses of sublingual buprenorphine. The clinician notes that the patient's overall progress indicates a positive response to the buprenorphine implant treatment, showcasing effective control over withdrawal symptoms and cravings. The patient herself expresses satisfaction with her experience.

## Case report 4

### Clinical case description

The patient, a 53-year-old male of African Italian ancestry, reported that his initial exposure to drugs, particularly THC, occurred around the age of 12. Subsequently, following the dissolution of his marriage, he had encounters with cocaine and later opioids, leading to the development of addiction.

### Traditional opioid agonist therapy

After a period spent abroad, the patient returned to Italy in 2002 and sought treatment from various Addiction Services, where he began treatment with methadone. Approximately four years ago he transitioned to OAT with sublingual buprenorphine. Upon admission to our Service in May 2022, his therapy consisted of sublingual buprenorphine 6 mg + sublingual naloxone 1.5 mg per day.

#### Psychological aspects prior to buprenorphine implant

During the meetings, the patient consistently demonstrated willingness and motivation. While his language was partially fluent, there were occasional interruptions attributed to difficulties in recalling certain phases of his life history. He showed spontaneity and did not need to be triggered to express himself, showing reflexivity and ability to contextualise. Adequate introspection and the absence of emotional blocks related to traumatic experiences were evident. The patient exhibited an internal locus of control and a sense of self-efficacy overall. From the behavioural point of view, within the service and with the clinical staff, we can highlight a good adherence to the indications given and to the scheduled appointments, and a good general compliance.

### Buprenorphine implant

Due to pharmacological stability for over 5 years and restricted drug use limited to cannabinoids, the patient was deemed eligible for the buprenorphine implant, meeting the psychosocial inclusion criteria. Following the proposal, he exhibited heightened curiosity about the implant, experiencing a sense of "euphoria" in anticipation of this novel experience. His interest increased during the presentation of the implant procedure, which he quickly accepted. The impetus to accept the proposal stemmed from some of the patient's reflections, especially regarding the potential for a lifestyle change and the reclamation of "his time," envisioning more opportunities for hobbies, family, and travel. Moreover, he imagined the recovery and achievement of life goals linked both to everyday life and to the possibility of planning without “personal” constraints of time and organisation. Eventually, some reflections "almost of tiredness" emerged, referencing both to the regular visits to the Addiction Service and to the interactions with other service users. This weariness stemmed from the perceived hindrance of traditional OAT, seen as a substantial impediment to daily freedom due to the commitment required for therapy. Additionally, it extended to the challenges in achieving personal and life goals.

Since this was the first buprenorphine implant carried out at our facility, it was necessary to draw up a procedure, and have it approved by the Health Management. This protocol encompassed the establishment of a dedicated outpatient file and the provision of a specialized room, serving both for the surgical procedure and for consultations with prospective candidates, some of whom were referred from other Addiction Services.

#### Psychological aspects following the buprenorphine implant

The patient exhibited a comprehensive shift in mood, a heightened inclination toward openness with others, and a rejuvenated approach to life planning. Following the implant procedure, the patient demonstrated improved speech fluency attributed to heightened introspective abilities. He identified the socio-affective dimension as the most significant element in the initiated change, leading to increased stability on the affective level. This translated into a newfound capacity to navigate relationships with more meaningful and secure emotional grounding. Moreover, the initial days following the implant marked a shift in self-perception and how the patient was perceived by others. The awareness of the significant impact of the intervention on his life became apparent, bringing about a rediscovery of energy, an enhanced "esprit de vivre", and a transformation in interpersonal relations with the Addiction Service staff. Overall, a newfound optimism and fortitude was evident.

#### Follow-up visits

During the post-implant interviews, the patient was subjected to a patient-reported outcome (PRO) measure using a visual analog scale (VAS) to capture the severity or other aspects of craving. A VAS measure usually requires participants to indicate their response by marking a point on a 100-mm line, with the extremities represented by 0 as "no craving for heroin" and 100 as "absolute craving for heroin" [12, 14]. At the follow-up the patient reported a “lack of craving” in terms of intensity and frequency, and he also denied the possibility of starting drug use in the event of experiencing craving. Throughout the course of treatment, the patient underwent weekly visits during the first month, followed by fortnightly visits in the second month, and eventually transitioning to monthly visits. Toxicological tests were conducted during these visits to monitor the patient's progress. No additional sublingual buprenorphine tablets or other drugs were necessary. Out of 11 toxicological tests carried out on urine samples, 2 were negative for all the substances sought. All other tests showed positivity for cannabinoids; this was consistent with the patient’s reported reduced daily use of THC before going to sleep.

From the outset, the patient expressed a reluctance to pursue a second implant, although he did not entirely rule out the possibility. As a result, the decision was made to defer the removal of the implant, allowing for close follow-up to monitor any changes. If needed, oral therapy could be resumed while awaiting a potential second implant to be grafted. In line with the patient's preferences and the agreement with healthcare providers, the implant remained in place beyond the initially planned sixth month. This extension allowed the patient additional time to contemplate the option of a second implant while ongoing urine buprenorphine screening, toxicological monitoring, and regular interviews were conducted. The removal was originally scheduled for the end of the seventh month. However, due to the patient's unavailability, primarily driven by severe personal reasons, the removal was subsequently postponed by two weeks. As of today, the removal procedure has been successfully performed and the patient exhibits a complete absence of craving and no desire to use substances. During the last interview the patient reported: *“Every day I feel better!”*.

## Conclusions

Overall, the patient has experienced significant improvements in mood, interpersonal openness, and life planning. Additionally, there appears to be a reduction in THC use. Remarkably, even after the removal of the implant, the patient has not reported any cravings related to substance use.

## Case report 5

### Clinical case description

The patient, a 40-year-old male of Caucasian Italian ancestry university graduate currently in permanent employment, initiated drug experimentation around the age of 20. In this period, he became fascinated with and started attending rave parties, leading to gradual experimentation (reported as "controlled") with illicit drugs including Afghan opium, eventually resulting in the development of an addiction disorder.

#### Toxicological history

The patient’s toxicological history indicated a pattern of polyaddiction, involving the use of cannabinoids, particularly hashish, since the age of 18. At the age of 21, he began attending rave parties, engaging in simultaneous and occasional consumption of various drugs such as cocaine, MDMA, amphetamines, LSD, and Ketamine. Subsequently, the patient transitioned from regular opium use to heroin after approximately two years.

### Traditional opioid agonist therapy

He continued his substance use until the age of 25, at which point he initiated treatment with sublingual buprenorphine at an Addiction Service. Upon admission, he had a diagnosis of OUD in protracted remission under treatment with partial OAT (buprenorphine in combination with naloxone), and concomitant depression. Throughout the course of treatment, the patient maintained a steady intake of buprenorphine/naloxone sublingual tablets at a fixed dosage of 2 mg/0.5 mg per day. Since his initial admission, he consistently reported challenges in discontinuing OAT. Specifically, he reported being able to endure the absence of the medication for a few days (a maximum of 4 days), but with the onset of anxiety and worsening cravings due to the non-use of buprenorphine, he would resume his daily 2 mg dose intake. Since initiating OAT, the patient reported abstinence from opium or heroin use. Despite maintaining a stable clinical picture, the presence of recurrent unsuccessful attempts to discontinue OAT prompted consideration for transitioning from sublingual to subcutaneous therapy. In August 2022, during a toxicology interview, the possibility of buprenorphine implant therapy was proposed to the patient.

#### Psychological aspects prior to buprenorphine implant

In conjunction with the pharmacological aspect of the new therapy, the patient concurrently received treatment with specific antidepressants. Additionally, he has actively participated in individual psychotherapy for a duration of two years and is presently engaged in group psychotherapy. The patient promptly made himself available and demonstrated willing adherence to the instructions provided by the medical staff, consistently attending his scheduled appointments. Notably, he exhibited overall good mentalisation and fair self-esteem.

### Buprenorphine implant

The patient initially exhibited moderate curiosity during the first interview introducing the buprenorphine implant. However, his interest in the proposed treatment escalated swiftly. This interest and curiosity stimulated thoughts about the prospect of embarking on a new lifestyle. Throughout the interviews, he conveyed that he embraced the proposal due to tiredness from the constant mood swings induced by traditional OAT, which required daily visits to the facility. These factors, coupled with other personal considerations, amplified his discomfort with commitment, hindering the overall pursuit of life goals.

After establishing a dedicated room at our facility, the patient was directed to the Addiction Service, where an external doctor from the hospital conducted the implant surgery. Following the surgery, we maintained continuous monitoring through both group psychotherapy and individual therapy sessions.

Throughout the course of treatment, the patient initially underwent weekly visits during the first month, followed by fortnightly visits in the second and third month, and eventually transitioning to monthly visits. During these regular check-ups, the patient underwent toxicological controls, and notably, no additional sublingual buprenorphine tablets were required.

#### Psychological aspects following the buprenorphine implant

After the implant procedure, the patient experienced mood stabilization, which he described as surprisingly positive. This positive change was openly shared by the patient within the therapy group. He demonstrated introspective ability, albeit stereotyped, aligning with the ideological and social models of his peer group. Following the implant there appeared to be a recognition of subjective aspects that he had not previously explored, potentially serving as a foundation for renewed self-awareness. Moreover, the patient exhibited rich and articulate language, along with good introspective and self-reflective ability, fair insight, and a proficient recall of his life history. Shortly after the implant, he conveyed his sense of liberation in an email, stating: *"…I am a free man…"*. In a group session, he elaborated on this feeling, expressing that he now perceives himself as "like everyone else," no longer dependent on the daily tablet, and experiencing mood fluctuations akin to any other individual.

#### Follow-up visits

The patient has been undergoing treatment for several months and reported only experiencing a headache in the initial days following the implant. Toxicological controls indicate positivity only for THC, as the patient has consistently used cannabinoids by smoking a "joint" in the evening to relax before going to sleep, with no intention of discontinuing this habit.

In post-implant interviews, the patient underwent the VAS test and reported a "lack of craving" both in terms of intensity and frequency. Furthermore, he expressed no inclination to initiate drug use in the event of experiencing cravings.

From the outset, the patient has made it clear that he had no intention of pursuing a second implant. Although he does not rule out the possibility entirely, his hope is to attain complete liberation from OAT and, more broadly, from drugs. This suggests a reasonably sound capacity for judgment on his part. Hence, the decision was made to defer the removal of the initial implant, utilizing the gradual reduction of the drug, and assessing how best to support the patient on his journey towards detoxification.

## Conclusions

The patient appears to be progressing well on the detoxification path, as evidenced by his expressed intention to refrain from further OAT after the removal of the implant. The patient's determination is a crucial factor in the success of the detoxification process. The absence of craving after the removal of the implant, along with the noted mood stabilization and positive treatment perception reported by the patient, are significant indicators contributing to the success of the patient's detoxification journey.

## Case report 6

### Clinical case description

Filippo (fictitious name), a 23-year-old male of Caucasian Italian ancestry, reflects on his childhood, describing it as “normal”. His father is portrayed as a diligent worker, while his mother is characterized as a pragmatic and less sentimental woman. As an intelligent child, Filippo sensed the weight of the expectations his mother had placed on him. During the transition to middle school, he experienced a loss in friendships, became apathetic, distracted, and spent most of his time playing video games, rarely venturing outside. However, there was an improvement in his social life and academic performance during high school, which led Filippo to enrol in university, where he also initiated a romantic relationship with a girl.

#### Toxicological history

In the summer of 2018, following his first year of university, Filippo started experiencing anxiety disorders, making it challenging for him to cope with his exams. Simultaneously, he found out that his girlfriend was using heroin and cocaine. In response, he decided to experiment with these substances. Initially, his usage was occasional and seemed "manageable", but Filippo rapidly developed both physical and mental addiction. Furthermore, he began using cocaine to counteract the effects of heroin. His drug abuse progressively escalated from occasional to daily, extending beyond social contexts to solitary moments. Filippo found himself trapped in a vicious cycle, marked by a constant need to soothe himself and promptly reactivate. His academic performance suffered, and financial resources were increasingly diverted towards substance abuse. Recognizing the severity of the situation, Filippo sought help from a psychiatrist-psychotherapist, who advised him to approach an Addiction Service. Although Filippo was not fully convinced, he perceived that seeking help was his only viable option. When he shared his predicament with his family, their initial response was a mix of anger and concern. However, that single conversation remained an isolated instance, and subsequently, they seemed to adopt an approach of denial, choosing not to acknowledge the reality of Filippo's struggles.

### Traditional opioid agonist therapy

Filippo initiated his treatment at the Addiction Service in January 2020 with a dosage of 2 mg of sublingual buprenorphine. This regimen was subsequently increased to 4 mg after a few weeks. Notably, Filippo demonstrated commendable adherence to the treatment regimen, attending interviews regularly and concurrently engaging in private psychotherapy. He ceased his heroin use immediately after commencing OAT, and he also managed to discontinue cocaine, with only a few relapses in October 2020. Subsequently, Filippo experienced improvements in mood, school performance, and social interactions. However, his main concern revolved around the prospect of discontinuing the daily tablet intake.

### Buprenorphine implant

In January 2022, after Filippo's previous doctor departed from the service, I had a clinical interview with Filippo. During this meeting, I suggested a questionnaire to assess the current state of his therapy and his interest in transitioning to newly available drug formulations. Filippo embraced the idea of transitioning to a subcutaneous buprenorphine implant with enthusiasm. Despite considering the possibility of balancing his personal life with regular visits to the Addiction Service, he expressed a keen interest in the new treatment. At that point, he had been on a 4 mg sublingual buprenorphine tablet regimen for approximately two years, and his toxicological tests consistently showed negative results for illicit drugs.

Filippo's excitement stemmed from several profound considerations: the weariness of identifying himself as an addict, a label that he felt no longer accurately portrayed his current state; the conscious desire to disengage from the daily ritual of medication, which he defined as a "substitute" for his previous heroin use, and thus corresponded to him as if still "getting high" every day; and the wish to regain control over his daily routine without being tethered to the demands of therapy, envisioning a future where he could plan vacations and travel abroad without the constraints of regular visits. Lastly, Filippo held a hopeful anticipation of achieving a definitive conclusion to his therapy, marking a significant milestone in his journey towards recovery. Despite receiving comprehensive information from the data sheet, Filippo's determination to pursue the subcutaneous buprenorphine implant treatment remained unwavering. He maintained a steadfast commitment to this choice, eagerly anticipating further details about the practicality and feasibility of undergoing the implant procedure. The Addiction Service practitioners collaborated closely with the hospital pharmacy and the Palliative Care operating unit to efficiently organize the day of the surgery. On the morning of the surgery, Filippo exhibited no signs of agitation. He adhered to the given directions and refrained from taking the morning sublingual buprenorphine tablet. Without experiencing any withdrawal symptoms, he maintained focus on the day's objective. The surgical procedure proceeded smoothly, lasting approximately an hour, after which Filippo proceeded to attend his university activities.

#### Follow-up visits

In the days following the surgery, Filippo reported a sustained, almost heightened sense of well-being, exceptional concentration (especially in his studies), and an energy level he had not experienced before. While there may have been a brief, two-day period resembling a hypomanic phase, Filippo soon returned to a stable and regular state of well-being, seamlessly resuming his daily activities. Despite being aware of the option to supplement the implant with buprenorphine tablets, Filippo never felt the necessity to do so. In agreement with the department director, we limited Filippo's visits to the Ser.D to the bare minimum needed to perform the monitoring required by the implant protocol. These included urine tests at various intervals post-intervention: 1 week, 2 weeks, 1.5 months, 3 months, 4.5 months, 6 months, and 7 months. During these visits, we assessed his overall health, general well-being, reactions at the implantation site, degree of patient satisfaction, and any withdrawal or craving symptoms, along with potential drug abuse.

#### Psychological aspects following the buprenorphine implant

In our regular phone interviews with Filippo, he would describe positive events that were taking place in his life. Approximately four months post-intervention, during an in-person interview, we delved into the impact of the implant on Filippo's lifestyle. A significant transformation was evident: his self-perception had undergone a complete shift. During the six-month period of the implant, Filippo encountered an emotional reconnection with his mother when he shared his experience with the subcutaneous treatment. Until then, his addiction had only been briefly mentioned within the family context, resulting in a negative outcome. This revelation left his mother surprised, astonished, and moved, but also visibly proud.

On a separate occasion, Filippo attended a party and unexpectedly spent the night away from home. He emphasized that he only realized the next day that such spontaneity would not have been possible without the implant. Without the need for daily tablets, he could participate freely without the fear of experiencing withdrawal symptoms the following morning. He no longer needed the “daily heroin substitute” and he no longer needed heroin. These and other episodes strengthened his conviction to “get rid” of therapy and of the fear of not being able to “walk without that crutch”. His determination grew, accompanied by the belief that the removal of the implant would mark the conclusion of his therapy. Filippo explicitly requested the removal of the implant not at the initially specified deadline but at a later time, and he duly signed a written request expressing this desire.

## Conclusions

The implant removal occurred in mid-November 2022, precisely 7 months and 9 days after its initial placement. Despite Filippo was at the time a little tense, the removal proceeded smoothly. The urine test conducted at this time still showed a positive result for buprenorphine. In the subsequent days, Filippo experienced symptoms including chills, tearing, arthralgia, and asthenia. Initially attributing these symptoms to a form of flu without strong conviction, he persevered. After 20 days, despite lingering discomfort, his determination to discontinue oral OAT prevailed. The subsequent urine test confirmed the absence of buprenorphine, marking the achievement of Filippo's goal.

## General discussion

The buprenorphine implant represents an innovative formulation for OAT, specifically designed for individuals with OUD who have achieved stabilization through prior oral therapy. Notably, the implant demonstrates equivalent therapeutic effectiveness and similar rates of adverse effects when compared to standard sublingual buprenorphine or buprenorphine/naloxone tablets [6, 8]. Nonetheless, a comprehensive risk–benefit evaluation has revealed several advantages associated with the subcutaneous buprenorphine implant in comparison to conventional OAT [10]. These benefits include enhanced treatment adherence, improved quality of life for patients, decreased likelihood of engaging in illicit opioid abuse, and a reduced risk of misuse or diversion [10]. These findings have been validated through the experiences of the first six patients in Europe who underwent the buprenorphine implant, as outlined in this case series. The report provides insights into the tangible effects of the buprenorphine implant on patients' quality of life and the achievement of therapeutic objectives, specifically focusing on abstinence from illicit drug abuse and the detoxification process.

Eligible patients were carefully assessed by the medical equipe in terms of clinical, psychological, and pharmacological status. All patients had refrained from using illicit drugs, were receiving low-dose sublingual buprenorphine (≤ 8 mg), demonstrated adherence to OAT and regular visits to the Addiction Service, and exhibited psychological stability. The heterogeneity observed in this group of patients stemmed from variations in sociocultural background, gender, age, duration of substance abuse history, length of the period of drug abstinence, and the specifics of their medical and pharmacological history, including the duration, dosage, and any prior OAT before transitioning to buprenorphine (Table 5).

**Table 5:** Demographic and patients’ characteristics

	Patients (*n* = 6)
Female/Male	1/5
Age (years, mean ± SD)	48 ± 14
History of drug abuse (years, mean ± SD)	19 ± 12
Employment	truck driver (*n* = 1), pensioner (*n* = 1), shop assistant (*n* = 1), laborer (*n* = 1), clerk (*n* = 1), university student (*n* = 1)
Medical history	nil (*n* = 4), renal papillary adenocarcinoma (n = 1), cryoglobulinemia (*n* = 1), bipolar disorder (*n* = 1)
Main substances of abuse	IV heroin (*n* = 6), cocaine (*n* = 1), alcohol (*n* = 1)
Maintenance therapy dosage (mg)	2 (*n* = 1), 4 (*n* = 2), 6 (*n* = 1), 8 (*n* = 2)
Concomitant therapies	antidepressants (*n* = 4), anticonvulsants (*n* = 1), antipsychotics (*n* = 1)
SL bup treatment (days)	30 ≤ x ≤ 4.015

*SD* standard deviation, *SL bup* sublingual buprenorphine

Buprenorphine implant emerges as a viable treatment alternative for diverse patient profiles, contingent upon achieving a certain level of pharmacological stability (≤ 8 mg), psychological well-being, and a documented recent history of drug withdrawal.

The reaction of the patients to the implant proposal ranged from moderate interest in some cases to genuine enthusiasm in others as delineated in Table 6 (Buprenorphine implant proposal). All patients embraced the buprenorphine implant to enhance their quality of life, eliminating the need for regular visits to the Addiction Service for the administration of tablets and moving closer to complete detoxification.

**Table 6:** Main points emerged from the case series

	Main points emerged
Buprenorphine implant proposal*	• Psychological response to the proposal: moderate interest (*n* = 4), genuine enthusiasm (*n* = 2) • Motivation for the acceptance of the buprenorphine implant: achieving detoxification (*n* = 6), re-gain daily freedom (*n* = 6), improve quality of life (*n* = 6), reluctance to visit the Addiction Service and desire to evade the related self- and external-perceived stigma (*n* = 6), disengagement from daily intake and associated mood swing (*n* = 6)
Surgery outcome	• Intra-operative: smooth surgical procedure (*n* = 6) • Post-operative: mild infection at the implant site (*n* = 1), overdose/withdrawal symptoms (*n* = 1)
Follow-up visits	• Duration: 3 months (case report 1) or 7–9 months • Frequency: variable from once a week to once every 1.5 months • Main findings and assessments: consistently negative opioid toxicological tests (*n* = 6), positive or very positive outcomes emerging from psychometric tests for cravings (*n* = 6), positive or very positive outcomes emerging from self-reported quality of life questionnaires (*n* = 6)
End of therapy	• Psychological response to the implant: satisfaction and happiness (*n* = 6), perception to be on the right path toward detoxification (*n* = 6), shift in self-perception, enhanced emotional stability, heightened introspective ability, renewed energy, and stabilized mood (*n* = 3), absence of cravings (*n* = 6), declining a second implant and additional OAT, patients conveyed a feeling of having “successfully distanced themselves from addiction after many years” (*n* = 6), • Clinicians observed that all patients exhibited all the necessary conditions for a favourable overall outcome of the treatment

*The clinical and pharmacological status was evaluated for all patients (*n* = 6) prior to the proposal. Buprenorphine implant proposal was delivered through oral or written means (e.g., questionnaire)

**Fig. 1:**
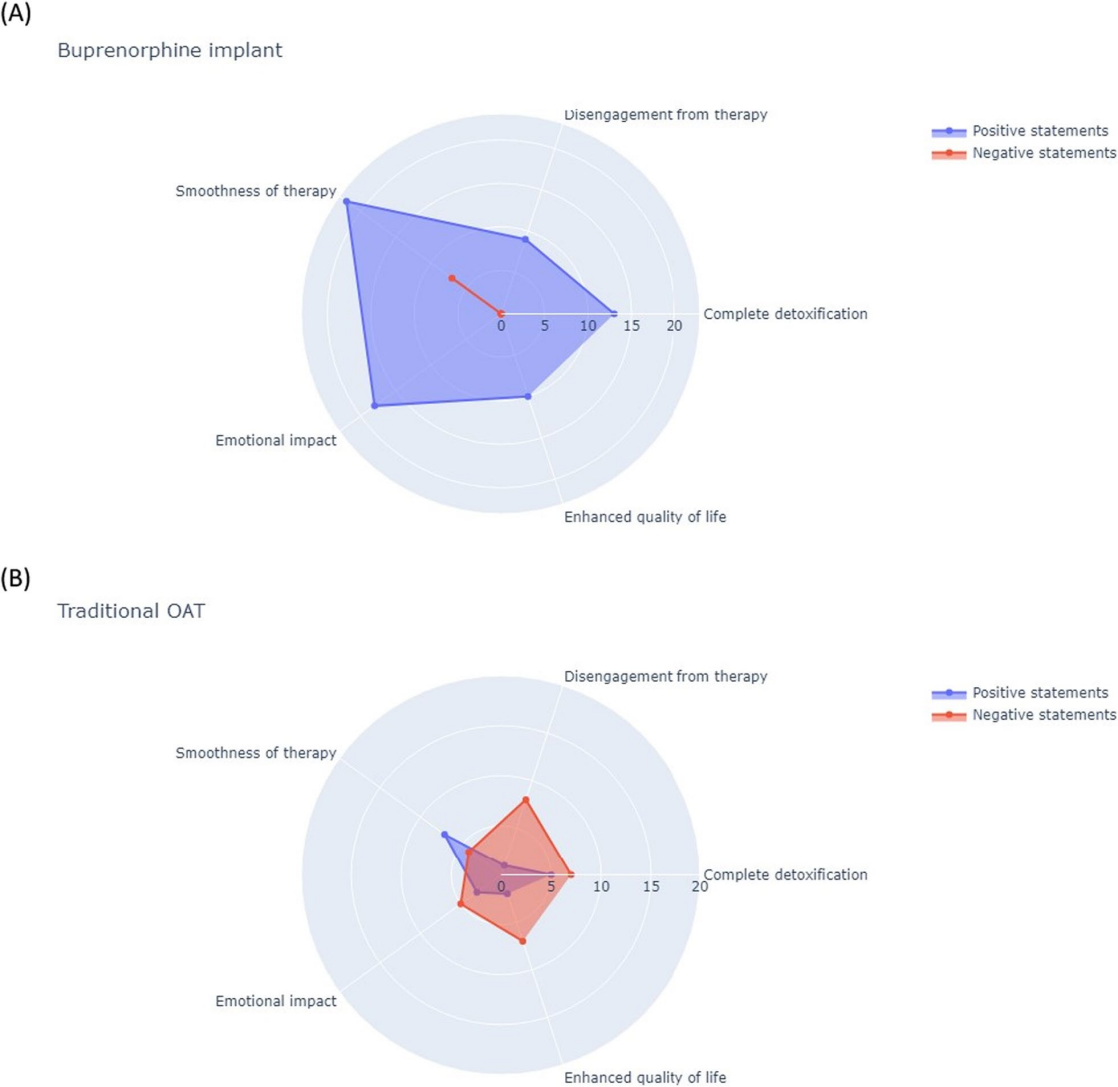
A narrative analysis of patients' reported experiences was conducted for both traditional OAT (**A**) and buprenorphine implant (**B**). The analysis was conducted by categorizing the statements related to each treatment into the five identified topics positioned at the vertices of the pentagon. The number of positive (blue line) and negative (red line) statements per topic were plotted along the direction of the corresponding vertex and connected by a 5-pointed closed line. The distance from the centre indicates the frequency of iterations. Notably, the scale of the pentagon differs between the two graphs

To assess the impact of both traditional OAT and buprenorphine implant, a semi-quantitative narrative analysis was conducted [15–18]. Every quote pertaining to patients' experiences with either treatment was considered in the analysis and subsequently categorised into one or more of the following topics: commitment to achieving complete detoxification, disengagement from therapy, smoothness of therapy, emotional impact, and improved quality of life in terms of free-time, finances, work, and interpersonal relationships (Fig. 1). Subsequently, the positive or negative valence associated with each statement was recorded. The implant was viewed as a valuable means to achieve abstinence from both drugs and medications, as evidenced by a total of 22 positive statements (Fig. 1A), compared to 6 for traditional OAT (Fig. 1B). The regular attendance at the Addiction Service was seen as a “constraint that disrupted daily routines” and “contributed to social stigma”, undermining patients' commitment to therapy and overall quality of life (7 out of 9 negative statements). The desire to break free from the daily tablet intake, perceived as a “substitute for heroin” and a source of mood swings, was a common sentiment. In contrast to the peaks associated with oral intake, the subcutaneous implant offered a stable release of buprenorphine, as evidenced by the 22 positive statements (Fig. 1A) compared to 7 (Fig. 1B) associated with traditional oral intake. This consistency helped in mitigating both physical and emotional fluctuations experienced by the patients.

In terms of surgical procedure, the buprenorphine implant insertion was carried out in a specialised facility, by a professional surgeon, and no significant issues were encountered for any of the patients (Table 6, Surgery outcome). Solely one patient developed a minor infection at the implant site, which was promptly addressed with antibiotics. He also reported a subjective feeling of overdose in the initial days post-insertion, followed by mild withdrawal symptoms, that were stabilised by a 3-day course of 1 mg sublingual buprenorphine. Consistent with findings from a previous study [19], the buprenorphine implant insertion procedure and the subsequent adaptation to treatment appear to be overall safe and well-tolerated.

During 6 months of follow-up, as outlined in Table 6 (Follow-up visits), the potential onset of withdrawal symptoms was closely monitored through regular assessments for most patients. Psychometric tests were also conducted to evaluate various aspects. Importantly, no patient reported experiencing cravings throughout the course of treatment, and all toxicological tests yielded negative results for the detection of illegal opioid abuse. All the patients expressed satisfaction with the buprenorphine implant treatment, and most of them reported being content with their decision, as indicated in Fig. 1 and Table 6 (End of therapy). On an emotional level, all patients reported a sense of well-being, with 18 positive statements compared to 3 positive statements for traditional OAT (Fig. 1A, B), and no instances of relapse were noted. Half of the patients experienced increased lucidity, improved introspective ability, and greater stability on the affective level (Table 6, End of therapy). The majority showed an on-axis mood (4 out of 6), absence of anxiety, hypnic pattern within limits, and restful sleep. Two out of six patients explicitly described a marked improvement in self-perception during the 6-month buprenorphine implant treatment. Overall, buprenorphine implant was perceived as a step closer to complete detoxification with 13 positive statements (Fig. 1A) vs. 5 positive statements for traditional OAT (Fig. 1B).

The removal procedure was successful for most of the patients, and none of them opted for a second implant. Solely one patient reintroduced sublingual buprenorphine at low dosages, although this decision was not prompted by any withdrawal symptom. Most importantly, none of the patients experienced craving episodes, indicating the potential for them to continue living without any OAT and ultimately achieve complete detoxification.

In summary, this case series explores the pioneering use of buprenorphine implant as a treatment option for OUD in a small European cohort of eligible patients. The findings suggest positive outcomes, including improved patient satisfaction and quality of life, reduced stigma associated with regular clinic attendance, and perceived advantages in achieving opioid abstinence. However, certain limitations must be acknowledged, including the small sample size, the relatively short follow-up period, and the reliance on self-reported questionnaires to evaluate patients’ perspective and experiences. The relatively small yet heterogenous sample size, while providing valuable insights into how various patient profiles might respond to this treatment approach, could affect the generalizability of the findings to a broader population. Moreover, the variability in the frequency and duration of follow-up visits, while enabling to capture the moderate-to-long term effects of the treatment, limits the ability to assess longer-term outcomes. Furthermore, the study's reliance on self-reported questionnaires while focusing on patients’ perspective, might introduce the possibility of response bias. This could include an inclination to offer responses that align with social expectations or recall biases. Therefore, in future studies the adoption of standardized assessment tools will ensure consistency and facilitate more robust cross-study comparisons. Future research should prioritise larger cohorts, encompassing comparative analyses with traditional OAT, and long-term investigations to assess sustained efficacy and diverse dynamics of patient profiles. Collaborative efforts to standardize assessment protocols across facilities would further strengthen the reproducibility of research findings in this evolving field.

## Final conclusions

This case series outlines the therapeutic journey of the first six European patients who underwent buprenorphine implant therapy. The results demonstrate favourable outcomes, including successful opioid abstention, alleviation of withdrawal symptoms, and enhanced quality of life and psychological well-being. Importantly, the treatment exhibited a high level of safety and tolerability, with no significant adverse events reported during the peri-operative period. The smooth insertion procedure and subsequent adaptation highlight the consistent benefits of the implant, with most patients achieving complete abstention, a milestone that might have been challenging with traditional approaches. Overall, the patients' satisfaction with the buprenorphine implant underscores its potential as a viable treatment option for pharmacologically stable individuals seeking to transition from traditional OAT. Nevertheless, further research into patient profiles, craving dynamics, and patient-centred outcomes is essential for optimizing personalized interventions in the field of addiction medicine.

## Supplementary Information

**Additional file 1: Case report 1.** Buprenorphine implant procedure.

**Additional file 2: Case report 2.** Buprenorphine implant procedure.

## Data Availability

The datasets used during the current study are available from the corresponding author on reasonable request.

## Abbreviations

OUD: Opioid use disorder
OAT: Opioid agonist therapy
HCV: Hepatitis C Virus
MASVE: Centro Manifestazioni Sistemiche Virus Epatitici, Systemic Manifestations of Hepatitis Virus Centre
WHOQOL-BREF: World Health Organization Quality of Life-BREF
THC: Tetrahydrocannabinol
VAS: Visual analog scale
MDMA: Methylenedioxymethamphetamine
LSD: Lysergic acid diethylamide

## References

[1] Dydyk AM, Jain NK, Gupta M. Opioid Use Disorder. [Updated 2023 Jul 21]. In: StatPearls [Internet]. Treasure Island (FL): StatPearls Publishing; 2024. https://www.ncbi.nlm.nih.gov/books/NBK553166/.

[2] Strang J, Volkow ND, Degenhardt L, Hickman M, Johnson K, Koob GF, *et al*. Opioid use disorder. Nat Rev Dis Primers. 2020;6(1):3. 31919349 10.1038/s41572-019-0137-5

[3] Overview | Drug misuse in over 16s: opioid detoxification | Guidance | NICE [Internet]. NICE; https://www.nice.org.uk/guidance/cg52. Accessed 1 Dec 2022.

[4] Bell J, Strang J. Medication treatment of opioid use disorder. Biol Psychiatry. 2020;87(1):82–8. 31420089 10.1016/j.biopsych.2019.06.020

[5] Mannaioni G, Lugoboni F. Precautions in the management of opioid agonist therapy: from target population characteristics to new formulations and post-marketing monitoring—a focus on the Italian system. Drugs Context. 2023;12. 10.7573/dic.2023-2-6 PMC10470859 37664791

[6] Rosenthal RN, Lofwall MR, Kim S, Chen M, Beebe KL, Vocci FJ, *et al*. Effect of buprenorphine implants on illicit opioid use among abstinent adults with opioid dependence treated with sublingual buprenorphine: a randomized clinical trial. JAMA. 2016;316(3):282–90. 27434441 10.1001/jama.2016.9382

[7] Lagios K. Buprenorphine: extended‐release formulations “a game changer”! Med J Aust. 2021;214(11):534. 10.5694/mja2.51098 34028813

[8] Rosenthal RN, Ling W, Casadonte P, Vocci F, Bailey GL, Kampman K, *et al*. Buprenorphine implants for treatment of opioid dependence: randomized comparison to placebo and sublingual buprenorphine/naloxone. Addiction (Abingdon, England). 2013;108(12):2141–9. 23919595 10.1111/add.12315 PMC4669043

[9] Ling W, Casadonte P, Bigelow G, Kampman KM, Patkar A, Bailey GL, *et al*. Buprenorphine implants for treatment of opioid dependence: a randomized controlled trial. JAMA. 2010;304(14):1576–83. 20940383 10.1001/jama.2010.1427

[10] Osborne V, Davies M, Roy D, Tescione F, Shakir SAW. Systematic benefit-risk assessment for buprenorphine implant: a semiquantitative method to support risk management. BMJ Evid Based Med. 2020;25(6):199–205. 32094200 10.1136/bmjebm-2019-111295 PMC7691701

[11] Wesson DR, Ling W. The clinical opiate withdrawal scale (COWS). J Psychoactive Drugs. 2003;35(2):253–9. 12924748 10.1080/02791072.2003.10400007

[12] Boyett B, Wiest K, McLeod LD, Nelson LM, Bickel WK, Learned SM, *et al*. Assessment of craving in opioid use disorder: psychometric evaluation and predictive validity of the opioid craving VAS. Drug Alcohol Depend. 2021;229: 109057. 34794061 10.1016/j.drugalcdep.2021.109057

[13] World Health Organization. WHOQOL: Measuring Quality of Life [Internet]. 2012. https://www.who.int/tools/whoqol. Accessed 26 Jan 2024.

[14] Goodyear K, Haass-Koffler CL. Opioid craving in human laboratory settings: a review of the challenges and limitations. Neurotherapeutics. 2020;17(1):100–4. 31650431 10.1007/s13311-019-00791-8 PMC7007448

[15] Nolte K, Drew AL, Friedmann PD, Romo E, Kinney LM, Stopka TJ. Opioid initiation and injection transition in rural northern New England: a mixed-methods approach. Drug Alcohol Depend. 2020;217: 108256. 32947174 10.1016/j.drugalcdep.2020.108256 PMC7769168

[16] Lai J, Goldfine C, Chapman B, Taylor M, Rosen R, Carreiro S, et al. Nobody wants to be narcan’d: a pilot qualitative analysis of drug users’ perspectives on naloxone. Western J Emerg Med. 2021;22(2). 10.5811/westjem.2020.10.48768 PMC7972385 33856321

[17] Meyer M, Rist B, Strasser J, Lang UE, Vogel M, Dürsteler KM, *et al*. Exploring why patients in heroin-assisted treatment are getting incarcerated—a qualitative study. BMC Psychiatry. 2022;22(1):169. 35255853 10.1186/s12888-022-03814-5 PMC8903629

[18] Scurti P, Nunzi M, Leonardi C, Pierlorenzi C, Marenzi R, Lamartora V. The experience of buprenorphine implant in patients with opioid use disorder: a series of narrative interviews. Front Psychiatry. 2023;31:14. 10.3389/fpsyt.2023.1205285 PMC10501400 37720906

[19] Frost M, Bailey GL, Lintzeris N, Strang J, Dunlop A, Nunes EV, *et al*. Long-term safety of a weekly and monthly subcutaneous buprenorphine depot (CAM2038) in the treatment of adult out-patients with opioid use disorder. Addiction. 2019;114(8):1416–26. 31013390 10.1111/add.14636 PMC6771955

