# Correction: Patients’ perspectives on buprenorphine subcutaneous implant: a case series

**DOI:** 10.1186/s13256-025-05024-5

**Published:** 2025-02-06

**Authors:** Claudio Pierlorenzi, Marco Nunzi, Sabino Cirulli, Giovanni Francesco Maria Direnzo, Lucia Curatella, Sandra Liberatori, Annalisa Pascucci, Edoardo Petrone, Generoso Ventre, Concettina Varango, Maria Luisa Pulito, Antonella Varango, Cosimo Dandolo, Brunella Occupati, Roberta Marenzi, Claudio Leonardi

**Affiliations:** 1 UOS Patologie da Dipendenza D9 ASL Roma 2, Rome, Italy; 2 UOC Patologie da Dipendenza D8 ASL Roma 2, Rome, Italy; 3 UOS Terapia del Dolore ASL Roma 2 Ospedale S. Eugenio, Rome, Italy; 4 Servizio Dipendenze Casalpusterlengo, ASST Lodi, Lodi, Italy; 5 Tossicologia Medica, Azienda Ospedaliero Universitaria Careggi, Florence, Italy; 6 ASST Papa Giovanni XXII, Ospedale Di Bergamo, Bergamo, Italy; 7 Dipartimento Tutela Delle Fragilita ASL Roma 2, Rome, Italy

**Correction: Journal of Medical Case Reports (2024) 18:202** 10.1186/s13256-024-04483-6

Following publication of the original article [1], the authors received a request from colleagues for further clinical details and outcomes on a case they presented, to which the authors responded by providing information on the final steps and subsequent phases following the explant procedure.

For these reasons, and upon reviewing the text of the publication, the authors identified a typographical error that arose during the translation from Italian to English. Therefore, the authors consider it important to include a correction note.

The case we are referring to is Case Report No. 5, involving a 40-year-old male of Caucasian Italian ancestry, with a university degree, currently employed full-time on a permanent basis.

The typographical error was determined by a question asking why the patient had anxiety and cravings concurrently to buprenorphine intake.

The given sentence in the ‘Traditional opioid agonist therapy’ section currently reads:

Specifically, he mentioned being able to refrain from the medication for a few days (up to a maximum of 4 days). However, with the onset of anxiety and intensified cravings for buprenorphine, the patient resumed his daily intake of 2 mg.

The sentence within the ‘Traditional opioid agonist therapy’ section should instead read:

Specifically, he reported being able to endure the absence of the medication for a few days (a maximum of 4 days), but with the onset of anxiety and worsening cravings due to the non-use of buprenorphine, he would resume his daily 2 mg dose intake.

The sentence in the ‘Traditional opioid agonist therapy’ section has been indicated in this correction article and the original article [1] has been corrected.
