# Supplementary material for: Patients' perspectives on buprenorphine subcutaneous implant: a case series
Source: J Med Case Rep. 2024 Apr 6;18:202. doi: 10.1186/s13256-024-04483-6 (PMC10998295; doi:10.1186/s13256-024-04483-6)
Supplement: Supplementary file 1 — Additional file 1: Case report 1. Buprenorphine implant procedure. [file 13256_2024_4483_MOESM1_ESM.docx]

**Appendix SI**

**Case report 1**

*Buprenorphine implant procedure:*

- The internal doctor made contact with the surgeon who would have performed the intervention and physically accompanied the patient with the drug to the implant site.
- The external doctor who physically performed the procedure found some difficulties in inserting the rods and had to reposition one of the 4 rods because it was not implanted correctly with respect to the fan-shaped arrangement. However, the procedure turned out to be quite smooth and simple as well as without any further complications.
- Urine control, pre- and post-implantation toxicological examination with craving evaluation and COWS (Clinical Opiate Withdrawal Scale) were performed.
- Sublingual buprenorphine 1 mg was administered for 3 days post-operatively from day 5 to day 8.
