# Supplementary material for: Patients' perspectives on buprenorphine subcutaneous implant: a case series
Source: J Med Case Rep. 2024 Apr 6;18:202. doi: 10.1186/s13256-024-04483-6 (PMC10998295; doi:10.1186/s13256-024-04483-6)
Supplement: Supplementary file 2 — Additional file 2: Case report 2. Buprenorphine implant procedure. [file 13256_2024_4483_MOESM2_ESM.docx]

**Appendix SII**

**Case report 2**

*Buprenorphine implant procedure:*

- The internal doctor made contact with the surgeon who would have performed the intervention and physically accompanied the patient with the drug to the implant site. It was a relatively simple procedure.
- The external doctor who physically performed the procedure did not notice any difficulties in inserting the 4 rods.
- Urine control, pre- and post-implantation toxicological examination with craving evaluation and COWS (Clinical Opiate Withdrawal Scale) were performed.
